# Comprehensive study on *ERG* gene expression in normal karyotype acute myeloid leukemia: *ERG* expression is of limited prognostic value, whereas the accumulation of adverse prognostic markers stepwise worsens the prognosis

**DOI:** 10.1038/bcj.2016.120

**Published:** 2016-12-09

**Authors:** S Weber, T Haferlach, C Haferlach, W Kern

**Affiliations:** 1 MLL Munich Leukemia Laboratory, Munich, Germany

The clinical course of normal karyotype acute myeloid leukemia (CN-AML) is very heterogeneous and partly reflected by specific molecular abnormalities.^1^ The most useful markers implicated in prognostication are *FLT3* internal tandem duplication (*FLT3*-ITD), *NPM1* mutations (mut), biallelic *CEBPA*mut and *RUNX1*mut, with the latter three being now integrated in the updated WHO classification.^2, 3, 4^ Beside these, considerably more molecular alterations have been identified in CN-AML, the prognostic relevance of which is not as clear. Deregulated expression of *ERG* (ets-related gene) represents one of these alterations, since high *ERG* expression has been allocated to lower complete remission (CR) rates and shorter disease-free survival, event-free survival (EFS) and overall survival (OS) in some studies,^5, 6, 7^ whereas another study of Marcucci *et al.*^8^ only reported an adverse effect of high *ERG* expression on the achievement of CR and on EFS. Besides the prognostic value of single alterations, it becomes increasingly important to consider individual markers in their genetic context, as the prognostic impact of the aforementioned parameters may vary depending on the presence (or absence) of other molecular markers. The best validated example is represented by *NPM1*mut and *FLT3*-ITD, as only *NPM1*mut patients without *FLT3*-ITD (low-risk) have, in contrast to their *FLT3*-ITD positive counterparts, a comparatively better outcome and would therefore no longer benefit from allogeneic stem cell transplantation.^2, 9^ To refine risk-adapted models, the analysis of recently described molecular alterations in the light of other relevant molecular prognosticators is needed. The aim of the present study therefore was to reveal putative associations of altered *ERG* gene expression to other molecular alterations and to assess the impact of deregulated *ERG* expression on outcome, either alone and moreover in the context of the previously defined molecular alterations.

A total of 325 younger (<65 years) *de novo* CN-AML patients (169 female, 156 male; median age 53 years, range 18–65 years) were investigated. Of these, 295 patients received intensive treatment according to German standard AML protocols^10^ and were subject to prognostic analysis. The diagnosis was made according to World Health Organization criteria.^11^ Chromosome banding analysis was performed for all patients according to standard procedures. *ERG* expression was measured in 64 peripheral blood and 261 bone marrow samples for consistency with our previous analysis, in which the same patients had been characterized for *BAALC* expression. This previous study aimed at evaluation of the prognostic value of *BAALC* expression and did not include data on *ERG* expression.^12^ Alterations in *ASXL1*, *CEBPA*, *DNMT3A*, *FLT3* (ITD and mutations in the tyrosine kinase domain (TKD)), *IDH1*, *IDH2*, *MLL*, *NPM1*, *NRAS*, *RUNX1*, *TET2* and *WT1* were analyzed by either polymerase chain reaction, Sanger sequencing or an amplicon deep-sequencing approach. Further details on patient characteristics and the study methodology are provided in the Supplementary Material.

In diagnostic CN-AML samples, the expression of *ERG* varied within a wide range from 0.1 to 1008% *ERG*/*ABL1* with a median of 189%. First, we evaluated associations of *ERG* expression levels, as continuous variable, with patient characteristics and molecular markers. In terms of patients characteristics, only a slightly negative correlation of *ERG* expression levels to age was revealed (*r*=−0.235, *P*<0.001; Supplementary Table S1). Regarding molecular alterations, *ERG* expression levels were found to overlap between the different genetic subgroups. Nevertheless, substantial differences in mean *ERG* expression levels were revealed. Higher *ERG* expression levels were significantly associated with high *BAALC* expression, high *FLT3*-ITD to *FLT3*wt ratios (⩾0.5; further termed *FLT3*-ITD⩾0.5) and *WT1*mut as well as with the absence of *NPM1*mut and *IDH1*R132mut (Figure 1a). These results are consistent with published data in terms of *BAALC* and *FLT3*-ITD, though *ERG* has been analyzed as a categorical parameter in these previous studies.^6, 7, 8^ Regarding the molecular intermediate-risk group of *NPM1*wt or *FLT3*-ITD⩾0.5, mean *ERG* expression levels were significantly higher as compared with the low-risk group (Figure 1b). Thus, overall an association of unfavorable prognostic parameters with high *ERG* expression levels was observed.

Given the strong correlation of high *ERG* expression levels to high *BAALC* expression as well as to different molecular genetic alterations, we analyzed correlations of expression of both genes, *ERG* and *BAALC*, to molecular mutations grouped into functional biological categories. Again, expression levels of both genes were found to overlap between the different functional subgroups. Slightly higher *ERG* expression levels were found in patients harboring mutations in one of the myeloid transcription factors, *CEBPA* and *RUNX1*, as compared with the patients without these mutations (Figure 1b). Also for *BAALC*, higher expression levels were significantly related to a mutated status in the myeloid transcription factor group. Further, substantially lower *BAALC* expression levels were observed in patients harboring mutations in genes involved in DNA methylation, including *DNMT3A*, *TET2*, *IDH1* and *IDH2* (Supplementary Figure S1). Interestingly, aside from the strong correlation to *FLT3*-ITD neither *ERG* nor *BAALC* revealed significant correlation to the activated signaling/proliferation group (Supplementary Figure S1). Therefore, in case of *FLT3*-ITD, the specific single gene association seems more important than a correlation to activated signaling/proliferation in general.

The impact of different parameters on OS and EFS was assessed by Cox regression analyses. The prognostic value of *BAALC* expression as a categorical variable (defining high and low expressers at certain cutoff levels^7, 12, 13^) has been shown before and could be corroborated, when analyzing *BAALC* expression as a continues variable, using log transformed expression levels (Table 1). *ERG* expression levels as a continuous log transformed parameter did neither affect OS nor EFS. This is in line with the study of Diffner *et al.,*^14^ where *ERG* expression analyzed as a continuous parameter did not impact on survival, but opposes the aforementioned studies,^5, 6, 7, 8^ where *ERG* expression has been associated with outcome, when dichotomized at certain cutoff levels (median or 75th percentile). Therefore, we performed Kaplan–Meier analysis dichotomizing *ERG* expression at distinct cutoff levels. A significant correlation to shorter EFS and a trend toward inferior OS was observed for *ERG* expression levels above the median (Figure 1c). As *ERG* expression strongly correlates with *NPM1*wt and *FLT3*-ITD, we assessed the prognostic value in the respective low- and intermediate-risk groups. As anticipated, no differences in EFS and OS were observed, which contrasts previous studies.^5, 6^ On the other hand, we found *BAALC* expression to strongly impact on EFS and OS in the intermediate-risk group of *NPM1*wt or *FLT3*-ITD, when dichotomized at the median (further termed low or high *BAALC*, respectively; Supplementary Figure S2). This result provides important prognostic information as the patients with *NPM1* wildtype or *FLT3*-ITD and high *BAALC* expression rather reflect OS of the ELN intermediate II-risk group, whereas the respective low *BAALC* expressers resemble outcome of the favorable-risk group.^15^

To clarify whether the sole accumulation of prognostic markers—in contrast to the above-tested specific genetic context of *NPM1* and *FLT3*—worsens prognosis, we determined the number of independent adverse prognostic parameters for each patient and performed survival analyses (Table 1). We defined four subgroups according to the number of adverse prognostic factors, namely high *BAALC*, *FLT3*-ITD⩾0.5, *MLL*-PTD and *WT1*mut for EFS as well as *ASXL1*mut, high *BAALC*, *FLT3*-ITD⩾0.5, *MLL*-PTD and *WT1*mut for OS; with group A: no adverse marker, group B: 1 adverse marker, group C: 2 adverse markers, group D: 3 or 4 adverse markers as none of the patients harbored concomitant alterations in all five adverse prognostic factors. The distribution of the adverse markers within these subgroups is given in the Supplementary Figure S3. Kaplan–Meier analysis revealed a 3-year EFS of 52% in group A, 36% in group B, 26% in group C and 7% in group D and a 3-year OS of 72% in group A, 61% in group B, 35% in group C and 13% in group D (Figure 1d). For EFS, group B and group C did not differ significantly, whereas substantial differences were shown for all other comparisons. Regarding OS, significant differences were shown for all comparisons except for group A versus group B (Figure 1d). In particular, Cox regression analyses revealed that EFS and OS were remarkably related to the number of adverse prognostic parameters (for both *P*<0.001; HR: 1.54 and HR: 1.70 per unfavorable marker positive, respectively). Thus, EFS and OS differed according to the number of adverse prognostic markers, suggesting that a comprehensive screening of molecular genetic alterations provides additional information for risk assessment in CN-AML. Furthermore, we performed multivariate analysis with the numbers of unfavorable markers (*ASXL1*mut (only for OS), high *BAALC*, *FLT3*-ITD⩾0.5, *MLL*-PTD and *WT1*mut: 0 to 4 adverse prognostic markers) and age. Both parameters were independently associated with shorter EFS (for both *P*<0.001; HR: 1.70 per unfavorable marker positive, HR: 1.35 per decade) and OS (for both *P*<0.001; HR: 1.97 per unfavorable marker positive, HR: 1.51 per decade).

In conclusion, we found *ERG* expression levels to correlate with specific molecular alteration and moreover to impact on EFS and OS though this impact was dependent on other molecular alterations. Besides the assessment of *ERG* expression, we were able to demonstrate that both the pattern of molecular alterations as well as the number of independent adverse markers, namely *ASXL1*mut, high *BAALC*, *FLT3*-ITD⩾0.5, *MLL*-PTD, *WT1*mut, are relevant for risk stratification in CN-AML.

## Figures and Tables

**Figure 1:**
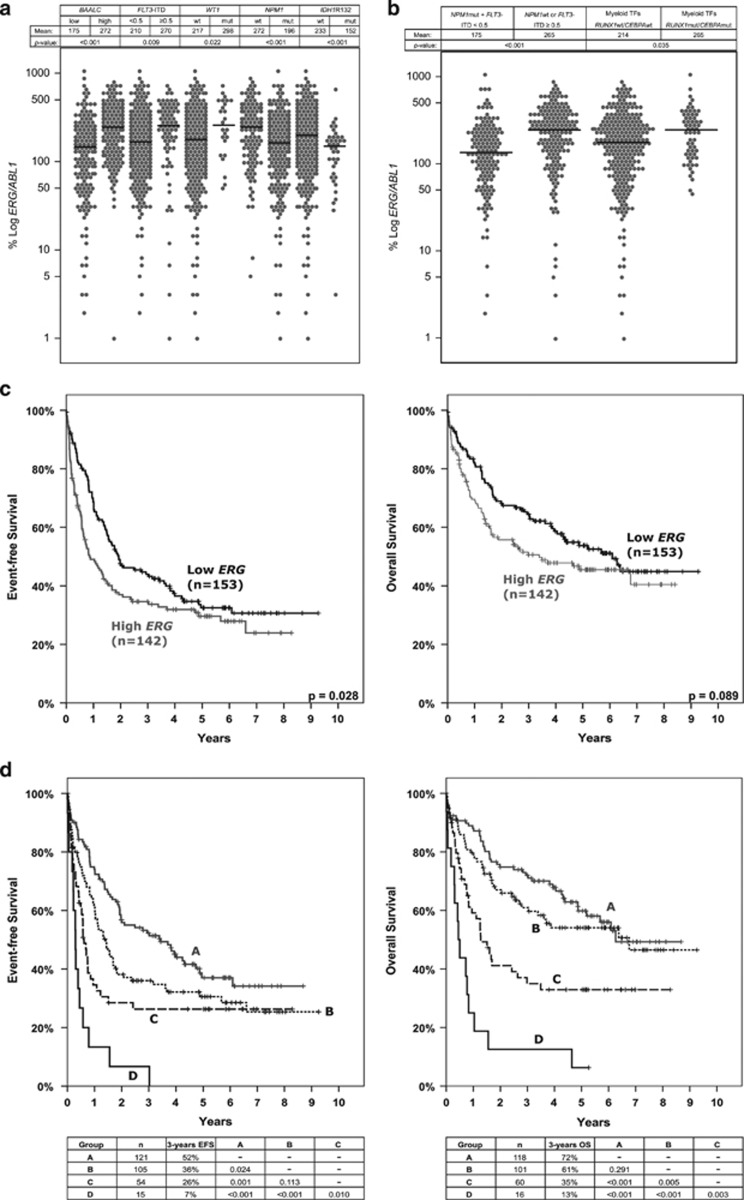
Associations of altered *ERG* gene expression to other molecular alterations (**a**, **b**) and survival analysis (**c**, **d**). Quantitative analysis showing *ERG* gene expression of the different subgroups of (**a**) concomitant molecular alterations and (**b**) molecular mutations grouped into prognostic or functional biological categories. Gray circles indicate single cases; black lines indicate mean expression. The *y*axis depicts the % *ERG*/*ABL1* on a logarithmic scale; the *x*axis depicts the different genetic subgroups. ITD, internal tandem duplication; TFs, transcription factors; mut, mutation; wt, wildtype. (**c**) Outcome of 295 intensively treated CN-AML patients aged younger than 65 years with respect to *ERG* expression. The median expression level was used to dichotomize the total patient cohort into low (black) and high (gray) *ERG* expressers. EFS at 3 years: Low *ERG*: 44% versus high *ERG*: 35%, *P*=0.028; OS at 3 years: Low *ERG*: 65% versus high *ERG*: 51%, *P*=0.089. (**d**) Outcome at 3 years in the four subgroups allocated according to the number of adverse prognostic markers: group A (no adverse marker), group B (1 adverse marker), group C (2 adverse markers) and group D (⩾3 adverse markers).

**Table 1:** Frequencies of molecular genetic aberrations and Cox regression analyses for overall survival and event-free survival

	*Frequency*	*Cox regression for overall survival*						*Cox regression for event-free survival*					
	*Intensively treated pts*	*Univariate*			*Multivariate*			*Univariate*			*Multivariate*		
	n*=295 (%)*	*HR*	P*-value*	*95% CI*	*HR*	P*-value*	*95% CI*	*HR*	P*-value*	*95% CI*	*HR*	P*-value*	*95% CI*
Age		1.38^a^	<0.001	1.21–1.56	1.53^a^	<0.001	1.34–1.73	1.26^a^	<0.001	1.12–1.40	1.39^a^	<0.001	1.24–1.55
*ASXL1*mut	4	2.39	0.012	1.21–4.72	2.47	0.012	1.22–4.98	1.86	0.046	1.01–3.43	–	n.s.	–
Log *BAALC* expression	–	1.27	0.009	1.06–1.52				1.32	<0.001	1.13–1.53			
High *BAALC* (median)	50	1.59	0.007	1.14–2.22	1.36	0.099	0.95–1.95	1.68	<0.001	1.27–2.24	1.44	0.024	1.05–1.97
*CEBPA*biallelic	6	–	n.s.	–				–	n.s.	–			
*DNMT3A*mut	45	–	n.s.	–				1.28	0.083	0.97–1.71			
Log *ERG* expression	–	–	n.s.	–				–	n.s.	–			
High *ERG* (median)	49	1.33	0.090	0.96–1.85				1.34	0.030	1.03–1.82	–	n.s.	–
High *ERG* (75th percentile)	32	–	n.s.	–				–	n.s.	–			
*FLT3*-ITD	36	1.65	0.003	1.18–2.30				–	n.s.	–			
*FLT3*-ITD (⩾0.5)	22	2.15	<0.001	1.50–3.08	2.28	<0.001	1.55–3.36	1.69	0.002	1.22–2.34	1.57	0.012	1.11–2.23
*NPM1*wt or *FLT3*-ITD	63	1.79	0.002	1.25–2.56				1.41	0.021	1.05–1.90			
*NPM1*wt or *FLT3*-ITD (⩾0.5)	53	1.79	0.001	1.28–2.52				1.60	0.001	1.20–2.13			
*FLT3*-TKD	10	–	n.s.	–				–	n.s.	–			
*IDH1*R132mut	12	–	n.s.	–				–	n.s.	–			
*IDH2*R140mut	13	–	n.s.	–				–	n.s.	–			
*IDH2*R172mut	2	–	n.s.	–				–	n.s.	–			
*MLL*-PTD	8	2.46	0.001	1.46–4.15	2.53	0.001	1.47–4.34	1.70	0.043	1.02–2.84	1.67	0.057	0.99–2.84
*NPM1*mut	64	–	n.s.	–				0.77	0.078	0.58–1.03			
*NRAS*mut	16	–	n.s.	–				–	n.s.	–			
*RUNX1*mut	10	–	n.s.	–				–	n.s.	–			
*TET2*mut	17	–	n.s.	–				–	n.s.	–			
*WT1*mut	9	1.95	0.010	1.18–3.25	2.57	0.001	1.46–4.52	2.18	0.000	1.41–3.38	2.47	<0.001	1.54–3.98

Abbreviations: CI, confidence interval; HR, hazard ratio; ITD, internal tandem duplication; mut, mutation; n.s., not significant; PTD, partial tandem duplication; Pts, patients; TKD, tyrosine kinase domain.

a Per 10 years of increase.
