# Supplementary material for: Comprehensive study on ERG gene expression in normal karyotype acute myeloid leukemia: ERG expression is of limited prognostic value, whereas the accumulation of adverse prognostic markers stepwise worsens the prognosis
Source: Blood Cancer J. 2016 Dec 9;6(12):e507–. doi: 10.1038/bcj.2016.120 (PMC5223155; doi:10.1038/bcj.2016.120)
Supplement: Supplementary Information [file bcj2016120x1.docx]

**SUPPLEMENTAL INFORMATION**

**Comprehensive study on *ERG* gene expression in normal karyotype acute myeloid leukemia: *ERG* expression is of limited prognostic value, whereas the accumulation of adverse prognostic markers stepwise worsens the prognosis**

# Patients and methods

1. Patients

All samples were referred to our laboratory for diagnostic assessment of AML between September 2005 and September 2012. AML was diagnosed according to the FAB and WHO classifications.^1, 2^ To the best of our knowledge all patients had de novo AML without any preceding malignancy or MDS. Before therapy, all patients gave their informed consent for scientific evaluations, after having been advised about the purpose and investigational nature of the study. The study was approved by the Internal Review Board of the MLL and adhered to the tenets of the Declaration of Helsinki.

1. Cytomorphology and cytogenetics

Cytomorphologic assessment was based on May-Grünwald-Giemsa stains, myeloperoxidase reaction, and non-specific esterase using alpha-naphtyl-acetate as described previously and was performed according to the criteria defined in the FAB and the WHO classifications.^1-3^ Cytogenetic studies were performed in all cases after short-term culture. Karyotypes, analyzed after G-banding, were described according to the International System for Human Cytogenetic nomenclature.^4^

1. Isolation of nucleic acid

Mononuclear cells from peripheral blood or bone marrow were separated by Ficoll-Paque density gradient. Either mRNA or RNA was extracted with MagnaPureLC mRNA Kit I (Roche Applied Science, Mannheim, Germany) or with MagNA Pure 96 Cellular RNA Large Volume Kit (Roche Applied Science). The cDNA synthesis from mRNA or RNA from an equivalent of 2.5-5 x10^6^ cells was performed using 300 U Superscript II (Life Technologies, Darmstadt, Germany) and random hexamer primers (Roche Applied Science) in a 50 µl reaction. Genomic DNA was isolated with QIAamp DNA Mini kit or with QIAsymphony DSP DNA Midi Kit (Qiagen, Hilden, Germany) following the manufacturer's protocol.

1. ERG determination and molecular analysis

Quantitative assessments of *ERG* gene expression was performed by use of the Applied Biosystems 7500 Fast Real Time PCR System (Life Technologies). The expression of *ERG* was normalized against the expression of the reference gene *ABL1* to adjust for variations in RNA quality and varying efficiencies of cDNA synthesis. *ERG* and *ABL1* expression was determined in a 20µl reaction using previously described primers and probes.^5^ Amplification was performed after initial incubation at 95°C for 1 minute in a 2-step cycle procedure (95°C, 15 sec and 60°C, 30 sec) for 40 cycles. Each sample was analyzed in duplicate. To calculate *ERG* and *ABL1* copy numbers, standard curves for both assays were generated in every run by 10-fold dilution series of 5 different plasmid concentrations.

Analyses for alterations in *ASXL1*, *CEBPA*, *DNMT3A*, *FLT3* (ITD and tyrosine kinase domain (TKD)), *IDH1*, *IDH2*, *MLL*, *NPM1*, *NRAS*, *RUNX1*, *TET2* and *WT1* as well as for expression of *BAALC* were described previously.^6-16^ For *CEBPA* the term “biallelic” was used for patients with one N-terminal and one bZIP gene mutation, since it has been published that these mutations are usually biallelic and no wildtype *CEBPA* is expressed in these cases.^17^ Samples showing only one mutation were referred as to monoallelic *CEBPA* group.

1. Statistical analysis

Dichotomous variables were compared between different groups using the Fisher’s exact test and continuous variables by Student’s t-test. Correlation coefficient was specified as Spearman’s rank correlation. Overall survival (OS) was the time from diagnosis to death or last follow-up. Event-free survival (EFS) was defined as the time from diagnosis to treatment failure, relapse, death, or last follow-up. Survival curves were calculated for OS and EFS according to Kaplan-Meier and compared using the two-sided log rank test. Cox regression analysis was performed for OS and EFS with different parameters as covariates. Results were considered significant at p<0.05 for univariate analyses and p<0.1 for multivariate analyses. Parameters which were significant in univariate analyses were included into multivariate analyses. All reported *p*-values are two-sided. No adjustments for multiple comparisons were performed. SPSS software version 19.0.0 (IBM corporation, Armonk, NY) was used for statistical analysis.

# Supplementary Figures

**Figure S1:** **Quantitative analysis showing *BAALC* and *ERG* gene expression of the different subgroups.** Gray circles indicate single cases; black lines indicate mean expression. A) The y-axis depicts the % *BAALC*/*ABL1* on a logarithmic scale; the x-axis depicts the different genetic subgroups. B) The y-axis depicts the % *ERG*/*ABL1* on a logarithmic scale. Abbreviations: ITD, internal tandem duplication; TFs, transcription factors; mut, mutation; wt, wildtype.

**Figure S2: Outcome in the intermediate-risk group of *NPM1*wt or *FLT3*-ITD with respect to *BAALC* expression.** The median expression level was used to dichotomize the total patient cohort into low (black) and high (gray) *BAALC* expressers. EFS at 3 years: Low *BAALC*: 51% vs. high *BAALC*: 26%, p=0.009; OS at 3 years: Low *BAALC*: 68% vs. high *BAALC*: 40%, p=0.019.

**Figure S3: Distribution of markers within the subgroups determined by the number of adverse prognostic markers.** Rows correspond to the depicted genes and columns represent individual patients. Cases presented with a mutation or high expression are colored in red and wild-type cases in gray. The three subgroups are colored in red (group B), gray (group C) and light gray (group D). A) Distribution of the adverse prognostic factors (high *BAALC*, *FLT3*-ITD≥0.5, *MLL*-PTD and *WT1*mut) for EFS. B) Distribution of the adverse prognostic factors (*ASXL1*mut, high *BAALC*, *FLT3*-ITD≥0.5, *MLL*-PTD and *WT1*mut) for OS. Abbreviations: ITD, internal tandem duplication; PTD, partial tandem duplication.

**Reference List**

1 Bennett JM, Catovsky D, Daniel MT, Flandrin G, Galton DA, Gralnick HR, et al. Proposals for the classification of the acute leukaemias. French-American-British (FAB) co-operative group. *Br J Haematol* 1976; **33**: 451-458.

2 Arber DA, Brunning RD, Le Beau MM, Falini B, Vardiman J, Porwit A, et al. Acute myeloid leukemia with recurrent genetic abnormalities. In: Swerdlow SH, Campo E, Harris NL, Jaffe ES, Pileri SA, Stein H, et al., editors. WHO Classification of Tumours of Haematopoietic and Lymphoid Tissues.Lyon: International Agency for Research on Cancer (IARC); 2008. p. 110-23.

3 Haferlach T, Kern W, Schoch C, Hiddemann W, Sauerland MC. Morphologic dysplasia in acute myeloid leukemia: importance of granulocytic dysplasia. *J Clin Oncol* 2003; **21**: 3004-3005.

4 McGowan-Jordan J, Simons A, Schmid M. ISCN 2016: An International System for Human Cytogenomic Nomenclature. Basel, New York: Karger; 2016.

5 Weber S, Haferlach C, Jeromin S, Nadarajah N, Dicker F, Noel L, et al. Gain of chromosome 21 or amplification of chromosome arm 21q is one mechanism for increased ERG expression in acute myeloid leukemia. *Genes Chromosomes Cancer* 2016; **55**: 148-157.

6 Schnittger S, Schoch C, Kern W, Mecucci C, Tschulik C, Martelli MF, et al. Nucleophosmin gene mutations are predictors of favorable prognosis in acute myelogenous leukemia with a normal karyotype. *Blood* 2005; **106**: 3733-3739.

7 Dicker F, Haferlach C, Kern W, Haferlach T, Schnittger S. Trisomy 13 is strongly associated with AML1/RUNX1 mutations and increased FLT3 expression in acute myeloid leukemia. *Blood* 2007; **110**: 1308-1316.

8 Grossmann V, Schnittger S, Schindela S, Klein HU, Eder C, Dugas M, et al. Strategy for robust detection of insertions, deletions, and point mutations in CEBPA, a GC-rich content gene, using 454 next-generation deep-sequencing technology. *J Mol Diagn* 2011; **13**: 129-136.

9 Schnittger S, Eder C, Jeromin S, Alpermann T, Fasan A, Grossmann V, et al. ASXL1 exon 12 mutations are frequent in AML with intermediate risk karyotype and are independently associated with an adverse outcome. *Leukemia* 2013; **27**: 82-91.

10 Bacher U, Haferlach C, Kern W, Haferlach T, Schnittger S. Prognostic relevance of FLT3-TKD mutations in AML: the combination matters--an analysis of 3082 patients. *Blood* 2008; **111**: 2527-2537.

11 Schnittger S, Haferlach C, Ulke M, Alpermann T, Kern W, Haferlach T. IDH1 mutations are detected in 6.6% of 1414 AML patients and are associated with intermediate risk karyotype and unfavorable prognosis in adults younger than 60 years and unmutated NPM1 status. *Blood* 2010; **116**: 5486-5496.

12 Bacher U, Haferlach T, Schoch C, Kern W, Schnittger S. Implications of NRAS mutations in AML: a study of 2502 patients. *Blood* 2006; **107**: 3847-3853.

13 Kohlmann A, Grossmann V, Klein HU, Schindela S, Weiss T, Kazak B, et al. Next-generation sequencing technology reveals a characteristic pattern of molecular mutations in 72.8% of chronic myelomonocytic leukemia by detecting frequent alterations in TET2, CBL, RAS, and RUNX1. *J Clin Oncol* 2010; **28**: 3858-3865.

14 Schnittger S, Kinkelin U, Schoch C, Heinecke A, Haase D, Haferlach T, et al. Screening for MLL tandem duplication in 387 unselected patients with AML identify a prognostically unfavorable subset of AML. *Leukemia* 2000; **14**: 796-804.

15 Schnittger S, Schoch C, Dugas M, Kern W, Staib P, Wuchter C, et al. Analysis of FLT3 length mutations in 1003 patients with acute myeloid leukemia: correlation to cytogenetics, FAB subtype, and prognosis in the AMLCG study and usefulness as a marker for the detection of minimal residual disease. *Blood* 2002; **100**: 59-66.

16 Weber S, Alpermann T, Dicker F, Jeromin S, Nadarajah N, Eder C, et al. BAALC expression: a suitable marker for prognostic risk stratification and detection of residual disease in cytogenetically normal acute myeloid leukemia. *Blood Cancer J* 2014; **4**: e173.

17 Wouters BJ, Lowenberg B, Erpelinck-Verschueren CA, van Putten WL, Valk PJ, Delwel R. Double CEBPA mutations, but not single CEBPA mutations, define a subgroup of acute myeloid leukemia with a distinctive gene expression profile that is uniquely associated with a favorable outcome. *Blood* 2009; **113**: 3088-3091.
