# Supplementary figures and images for: Comprehensive study on ERG gene expression in normal karyotype acute myeloid leukemia: ERG expression is of limited prognostic value, whereas the accumulation of adverse prognostic markers stepwise worsens the prognosis
Source: Blood Cancer J. 2016 Dec 9;6(12):e507–. doi: 10.1038/bcj.2016.120 (PMC5223155; doi:10.1038/bcj.2016.120)

Supplementary Figure S1

A

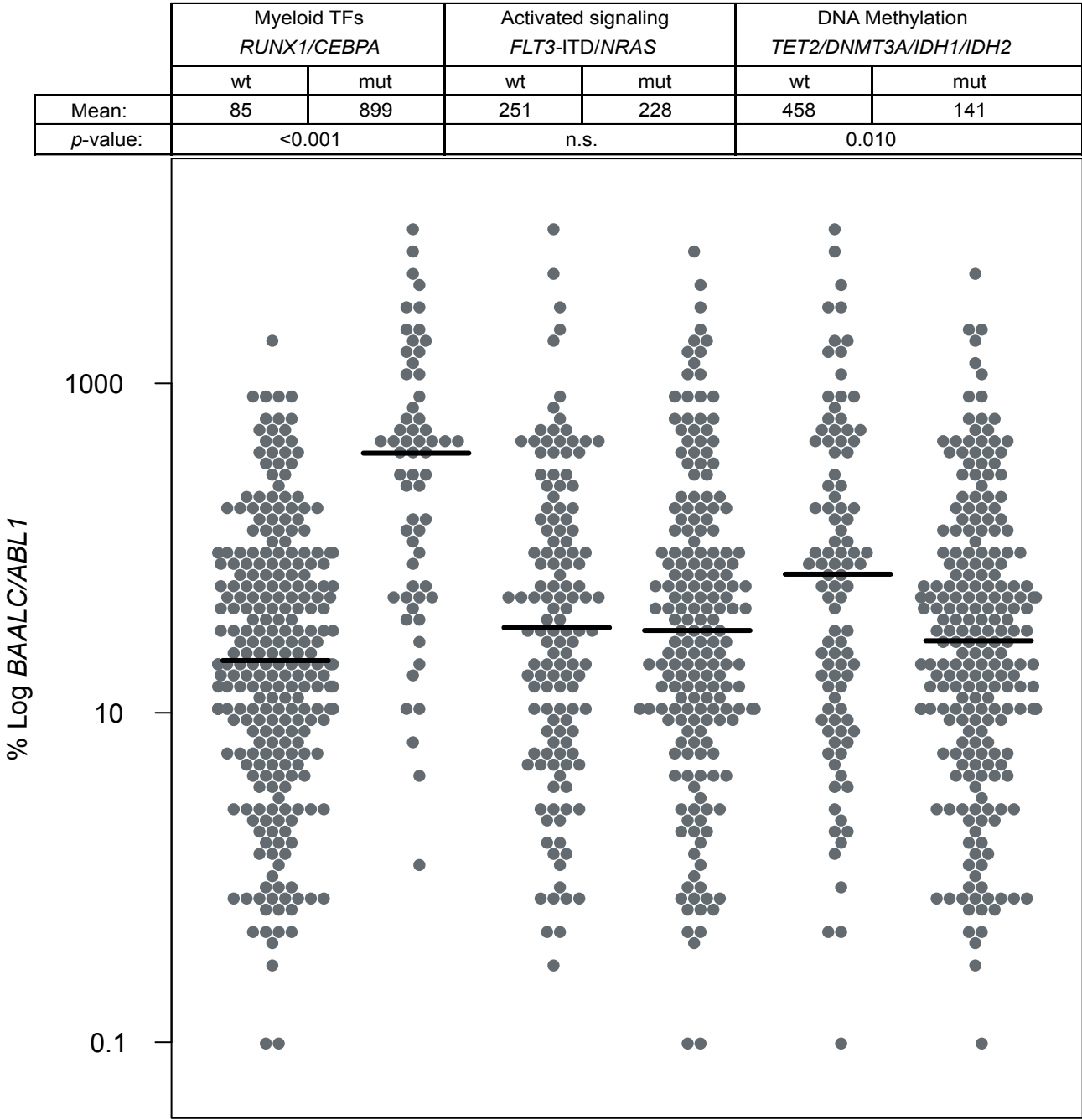

B

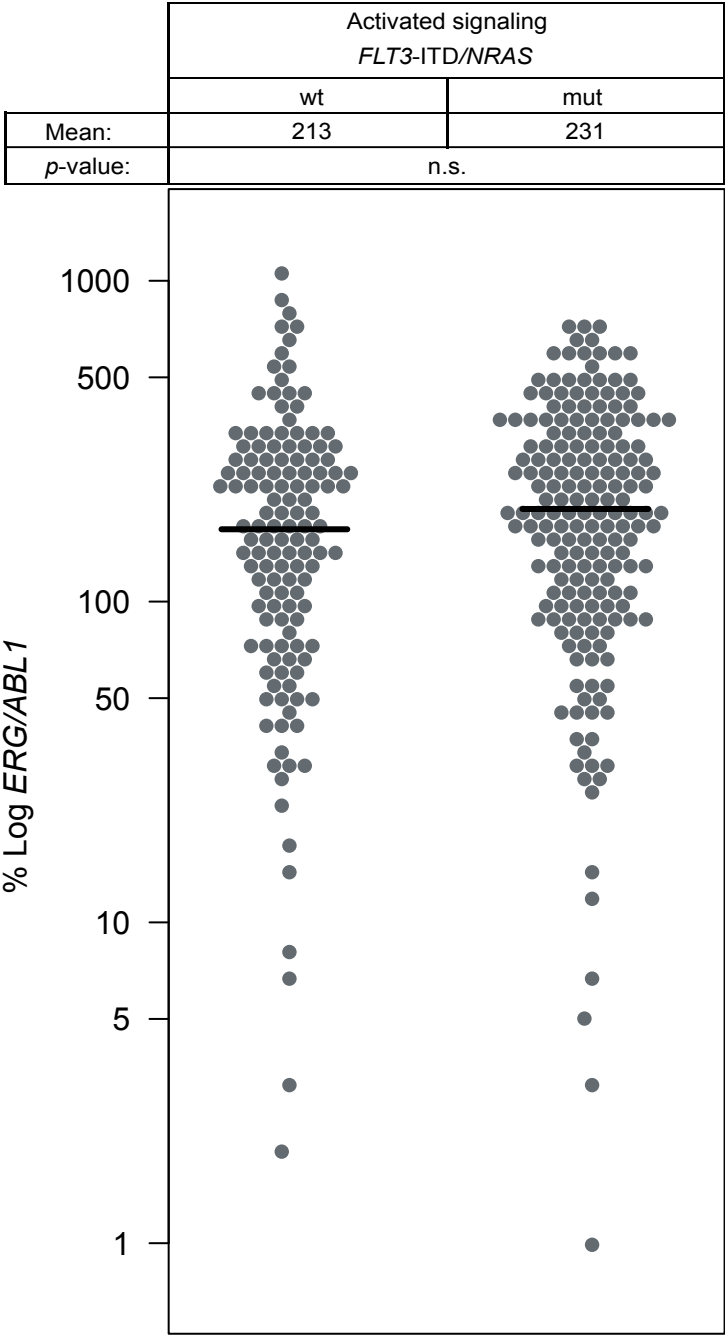

Supplement: Supplementary Figure S1 [file bcj2016120x3.pdf]

# Supplementary Figure S2

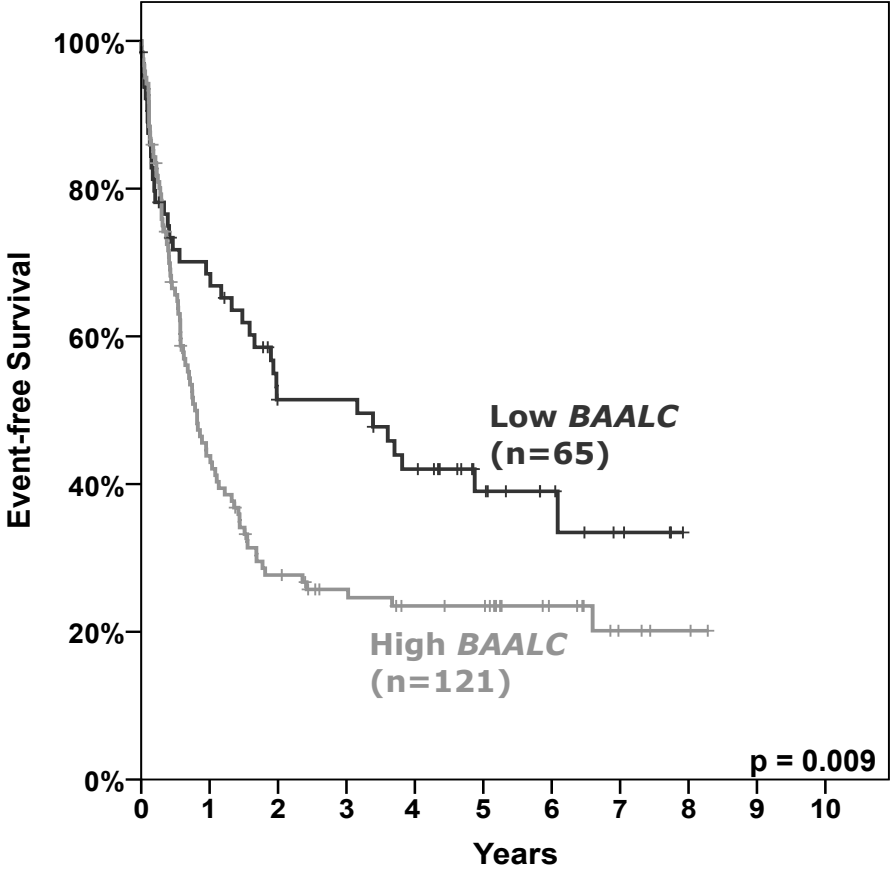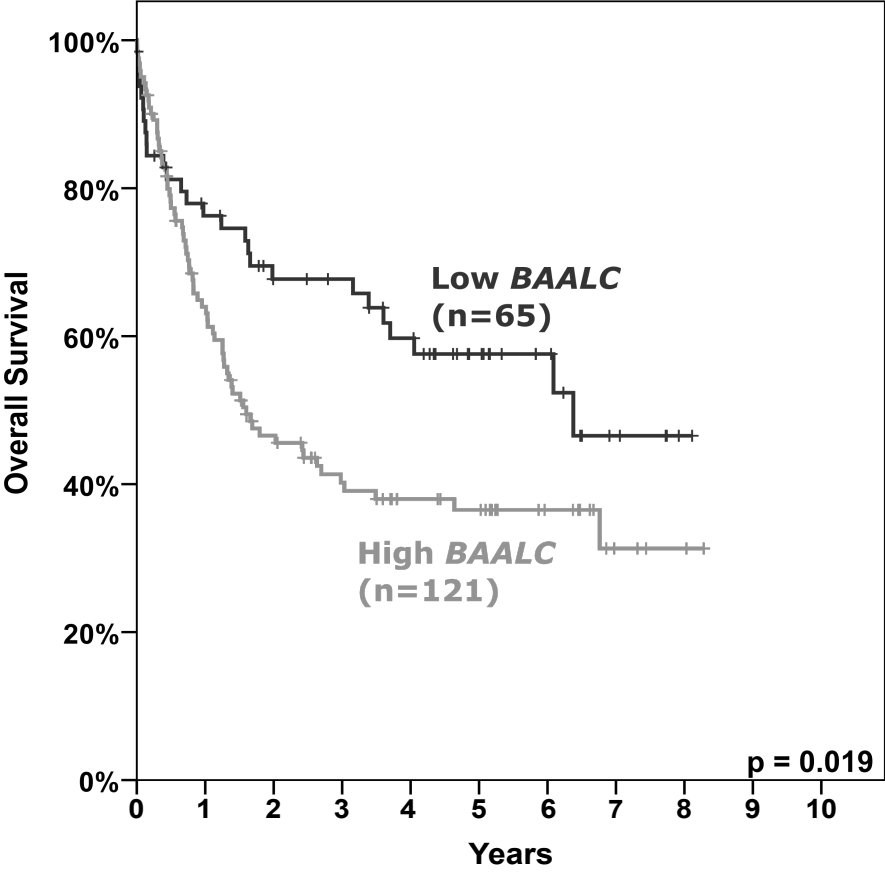

Supplement: Supplementary Figure S2 [file bcj2016120x4.pdf]

Supplementary Figure S3

A

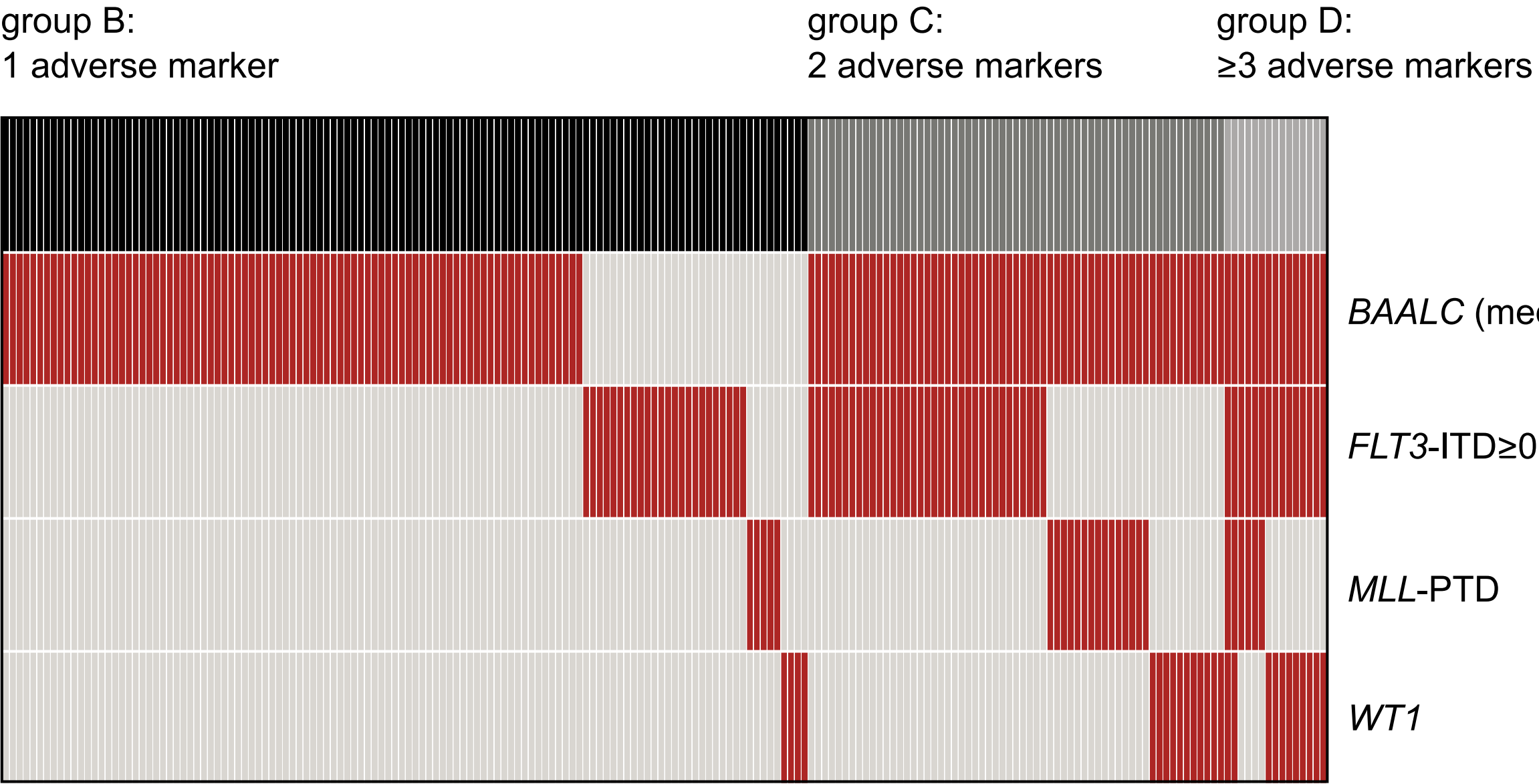

B

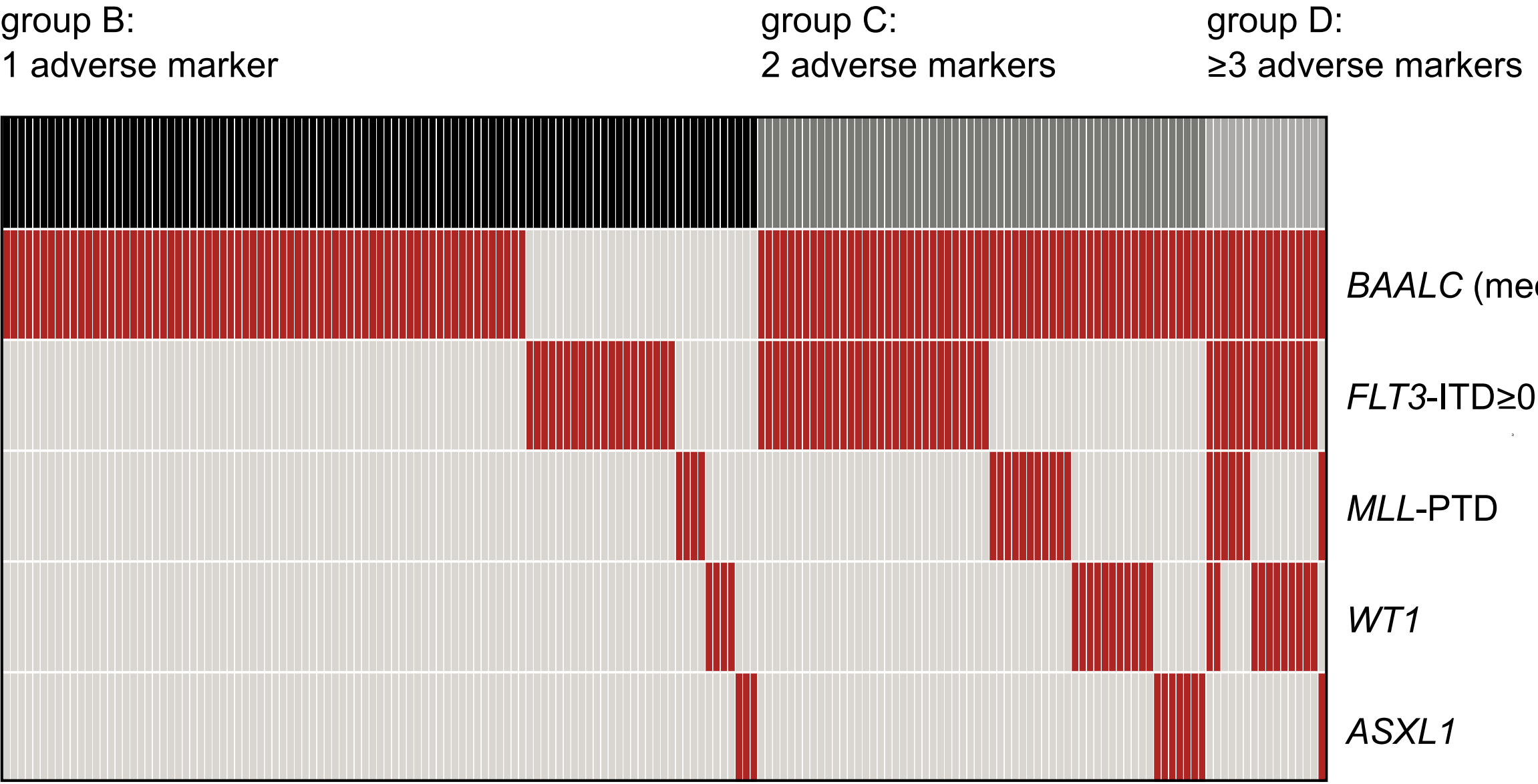

Supplement: Supplementary Figure S3 [file bcj2016120x5.pdf]
